# Spinal stabilization exercises for transversus abdominis and lumbar multifidus thickness via telerehabilitation and face-to-face approaches in patients with nonspecific chronic neck pain: a randomized controlled trial

**DOI:** 10.55730/1300-0144.5853

**Published:** 2024-07-12

**Authors:** Dilara ONAN, Erkan DEMİRCİ, Egemen TURHAN, Özlem ÜLGER

**Affiliations:** 1 Department of Physical Therapy and Rehabilitation, Faculty of Health Sciences, Yozgat Bozok University, Yozgat, Turkiye; 2 Spine Health Unit, Faculty of Physical Therapy and Rehabilitation, Hacettepe University, Ankara, Turkiye; 3 Department of Radiology, Memorial Hospital, Ankara, Turkiye; 4 Department of Orthopedics and Traumatology, Faculty of Medicine, Hacettepe University, Ankara, Turkiye

**Keywords:** Neck pain, transversus abdominis, lumbar multifidus, thickness, exercise, telerehabilitation

## Abstract

**Background/aim:**

Strengthening the muscles of the lumbar region in individuals with neck pain may be protective against future back problems. In addition, telerehabilitation applications, which gained momentum in the literature and clinical practice during the COVID-19 pandemic, are among the applications preferred by patients thanks to their various advantages. This study aimed to compare the effects of telerehabilitation and spinal stabilization exercises applied with face-to-face approaches on the thickness of the transversus abdominis (M.TrA) and lumbar multifidus (M.LM) muscles in patients with nonspecific neck pain.

**Materials and methods:**

The primary outcomes were the thickness of the M.TrA and M.LM. Neck pain intensity and neck disability were secondary outcomes. Muscle thickness was evaluated with an ultrasound device, neck pain intensity was assessed with a visual analog scale, and disability was assessed with the Neck Disability Index. Patients were randomly assigned to the telerehabilitation group (TRG) (n = 13) or the control group (CG) (n = 13). While the TRG did the exercises with live videos and video recordings, the CG did exercises face-to-face in the clinic. Both groups performed the same exercises for 45 minutes per session 3 days a week for 8 weeks.

**Results:**

At the end of the treatment, the thicknesses of the M.TrA and M.LM were increased and neck pain intensity and neck disability were decreased in both groups (p < 0.05). The groups were similar in terms of these variables (p > 0.05).

**Conclusion:**

Telerehabilitation and face-to-face spinal stabilization exercises are both beneficial for spinal muscle architecture and clinical variables as a preventive measure against future lower back problems in individuals with neck pain.

## 1. Introduction

When the healthy muscle structure is damaged due to various factors, the thicknesses and physiological cross-sectional areas of the muscles may decrease [1]. When the spine is considered as a whole chain within individuals with chronic neck pain (CNP), the posture of the entire spine may change and the lumbar region muscles may weaken as a result of the relationship between neck pain and low back pain [2]. Regarding the motor relationship between the cervical and lumbar spine regions, it has been shown that individuals with CNP have less motor control in the transversus abdominis muscle (M.TrA) during abdominal hollowing and rest compared to healthy controls [3]. It has also been stated that pain in the neck region may cause a greater response in lower spine regions such as the thoracic and lumbar regions [4]. In cases of orthopedic problems such as neck pain and low back pain, strengthening the muscles can increase the thicknesses and cross-sectional areas of the deep neck and back muscles, thereby improving spinal stabilization [5–7]. Therefore, the importance of exercise programs for strengthening the muscles emerges. It has been shown in different studies that spinal stabilization exercises (SSEs) targeting spine stabilization improve the architecture of the longus colli and cervical multifidus muscles in individuals with CNP [8–12] and the architecture of the M.TrA and lumbar multifidus muscle (M.LM) in individuals with low back pain [13,14]. However, considering that individuals with CNP are at risk of experiencing pain in the lumbar region in the future and that they have less motor control in M.TrA muscle activity according to the limited studies in the literature, the effectiveness of spine stabilization exercise programs on the thickness of lumbar region muscles is questionable. It is known that architectural features of the cervical and lumbar region muscles such as thickness can be improved with exercises [5–7]. Therefore, focusing exercise programs not only on the cervical region but also on the spinal region in individuals with CNP in clinics may reduce the risk of future low back pain.

Exercise programs can be applied face-to-face, as telerehabilitation, or as home exercise programs depending on requirements or preferences. The important point here is to follow the patients and provide feedback to ensure that the patients learn and apply the exercises correctly and that the exercise program achieves the intended outcomes and resolves the patients’ complaints [15]. The inability to monitor patients individually in home exercise programs is a frequently encountered limitation [15]. It is known that exercise programs applied face-to-face and with telerehabilitation are successfully performed by patients with spinal pain and high compliance is achieved [16]. In the present study, we considered two alternative hypotheses: H_0_ – In individuals with CNP, the effects of telerehabilitation and face-to-face SSEs on the muscle architecture of the lumbar region are similar; H_1_ – In individuals with CNP, the effects of telerehabilitation and face-to-face SSEs on the muscle architecture of the lumbar region are different. Thus, we aimed to investigate effects on the lumbar region muscle architecture by applying a SSE program both face-to-face and via telerehabilitation for individuals with nonspecific CNP for 8 weeks, following the patients individually.

## 2. Materials and methods

### 2.1. Trial design

In this randomized, controlled, parallel, and blindly evaluated study, the Consolidated Standard of Reporting Trials Statement (CONSORT) was applied. The CONSORT checklist is given in Table 1. Ethics approval was obtained from the Clinical Research Ethics Boards of Hacettepe University (2021/05-23, KA-20111) and the study was registered at ClinicalTrials.gov (NCT04691024).

Twenty-six patients met the inclusion criteria and were randomized with a 1:1 allocation ratio into the telerehabilitation group (TRG) and control group (CG). The study was conducted at the Spinal Health Clinic of the Faculty of Physical Therapy and Rehabilitation of Hacettepe University (Ankara, Türkiye).

### 2.2. Participants

Inclusion criteria were having a diagnosis of nonspecific neck pain lasting at least 3 months, being between the ages of 18 and 55 years, being literate, and being able to understand and complete the exercise program, reflected by a Montreal Cognitive Assessment Scale (MoCA) [17] total score of >21. The Turkish version of the MoCA has confirmed validity and reliability [18,19]. The possible score range of the MoCA is 0–30 points and scores of ≤21 indicate cognitive impairment in the Turkish population [18].

Exclusion criteria were having any cervical region diagnosis other than nonspecific neck pain, any systemic disease, pregnancy, acute fracture or infection, a history of surgery of the spine or upper extremity in the past year, and any other ongoing physical therapy and rehabilitation treatment.

Twenty-six patients met the inclusion criteria. These patients were divided into two groups with 13 in each group by randomization. The mean age was 35.76 ± 10.36 years in the TRG and 36.61 ± 9.99 years in the CG (36.19 ± 9.98 years for all patients), with no significant difference between the groups (p = 0.834). The female/male count was 9/4 in both the TRG and CG (18/8 in total) with no difference between the groups (p = 1.0).

The exercise program and assessments were conducted in the Spinal Health Clinic of the Faculty of Physical Therapy and Rehabilitation of Hacettepe University between March 2021 and June 2022. All assessments and exercises was performed by the same physiotherapist. The flow diagram including the study participants and randomization process are presented in the Figure.

### 2.3. Interventions

The TRG and CG completed the same SSEs and stretching exercises (the latter for 5 min before and after the SSEs). This SSE program was applied for both groups by a physiotherapist with a Master of Science degree for 45-min sessions 3 days a week for 8 weeks. The SSE program was administered with live video calls 2 days a week and recorded videos 1 day a week in the TRG. In the CG, the SEE program was applied as face-to-face exercises at the Spine Health Clinic. The difficulty level of the exercises was increased with each session and week (Figure S1).

### 2.4. Outcomes and objective assessment methods

#### 2.4.1.Primary outcome measures and objective assessment methods

The muscle thicknesses of the M.TrA and M.LM were the primary outcome measures. These muscle thicknesses were evaluated by rehabilitative ultrasound by a physiotherapist with a Master of Science degree who held rehabilitative ultrasound certification and had 2 years of relevant experience. Linear and convex probes (3.5–10 MHz, SonoStar Mobile Ultrasound Device, Guangzhou, China) were used for ultrasound assessment. The evaluations were performed with participants in supine resting position for the M.TrA and prone resting position for the M.LM at baseline and after the SSE program (Figure S2).

#### 2.4.2. Secondary outcome measures and objective assessment methods

Neck pain intensity and neck disability were the secondary outcome measures.

A visual analog scale (VAS) was used for pain intensity assessment. It had a horizontal line of 10 cm, with a mark of 0 representing no pain and 10 representing very intense pain. The patients marked their current levels of pain intensity as vertical lines on the horizontal line of the VAS [20].

Neck disability was evaluated with the Neck Disability Index (NDI). The NDI includes 10 items addressing pain intensity, personal care, lifting, work, headaches, concentration, sleeping, driving, reading, and recreation. Each item is scored with 0–5 points. Total scores of 0–4 points reflect no disability, 5–14 points signify mild disability, 14–24 points signify moderate disability, 25–34 signify severe disability, and 35–50 points signify complete disability [21,22].

### 2.5. Sample size

Initial power analysis was performed using G*Power software version 3.1. There are no previous studies on lumbar muscle thicknesses and SSE programs with CNP patients in the literature. Therefore, after 6 initial patients from the TRG and 6 initial patients from the CG completed the SSE program, the results of M.TrA muscle thickness during resting, as a primary outcome, were used for power analysis. For the difference between two independent means (i.e., two groups), a t-test was used with effect size of 1.34, alpha of 0.05, and power of 0.80. The mean M.TrA thickness during resting was 2.51 ± 0.57 for the TRG and 3.34 ± 0.66 for the CG. After calculations using these data, the total number of patients to be included in the study to ensure statistical power was a minimum of 20, with 10 patients each in the TRG and CG.

### 2.6. Randomization and blinding

Twenty-six patients met the inclusion criteria and were randomized into the TRG or CG with a 1:1 allocation ratio using a computer program with block size of four for the randomization. The patients in both groups were not informed about whether their group was the intervention group or control group. Therefore, patients were blinded because they did not know which group was the intervention group.

### 2.7. Statistical methods

Calculations and data analysis were performed by a blinded statistician. SPSS IBM Statistics 23.0 (IBM Corp., Armonk NY, USA) was used for the analysis and calculations. The Shapiro–Wilk test was used to determine the normality of the data. Values of percentage (%) and number (n) for categorical variables, mean and standard deviation for continuous variables, and median and minimum–maximum for noncontinuous variables were provided. Within groups, the paired sample t-test or Wilcoxon signed rank test was used for comparisons. Between groups, independent t-tests or Mann–Whitney U tests were used for comparisons. Effect size results were given as Cohen’s d. Values of p < 0.05 were accepted as statistically significant.

## 3. Results

Twenty-six patients were included in the study between March 2021 and June 2022. There were no differences between TRG and CG in terms of characteristics of the patients, as shown in Table 2, or baseline features of the outcome measures, as shown in Tables 3 and 4.

### 3.1. Results of primary outcomes: muscle thickness of the transversus abdominis and lumbar multifidus

At the end of the 8-week exercise program, there were improvements in right M.TrA resting and contraction, left M.TrA resting and contraction, and right and left M.LM in the TRG. Significant differences were found for right and left M.TrA resting and for right and left M.LM (p < 0.05 for all). There were similar positive changes in the two groups at the end of 8 weeks for all lumbar muscle variables (p > 0.05). Right-side M.TrA contraction showed more improvement compared to left-side M.TrA in the CG (p < 0.05). The thickness results for the M.TrA and M.LM are presented in Table 3.

### 3.2. Results of secondary outcomes: neck pain intensity and neck disability

Neck pain intensity and disability levels decreased in both the TRG and CG groups after the 8-week exercise program (p < 0.05). Neck pain intensity and disability both had large effect sizes. However, the effect of neck pain intensity was greater than 1 (i.e., very large) in both groups (for TRG: 3.34; for CG: 2.90). There were no differences between the groups after 8 weeks in terms of neck pain intensity or disability (p > 0.05), as shown in Table 4.

## 4. Discussion

This randomized controlled trial aimed to investigate the impact of a SSE program on the muscular architecture of the lumbar region. The program was administered to individuals with nonspecific CNP using both face-to-face and telerehabilitation methods over an 8-week period. Our findings indicated that the thickness of the muscles in the lumbar region increased while neck pain intensity and neck disability decreased after the exercise program in both groups. Despite the study being conducted amidst the conditions imposed by the COVID-19 pandemic, similar results were observed between the two groups for all evaluated outcome measures at the end of 8 weeks. This finding demonstrates that SSEs performed remotely via telerehabilitation are equally beneficial for lumbar region muscle architecture, neck pain intensity, and neck disability in comparison to the traditional face-to-face approach.

Telerehabilitation provides advantages in terms of not experiencing difficulty in reaching health professionals due to distance, being able to compensate for missing treatment sessions due to work or childcare responsibilities, reducing the financial costs of the treatment and waiting times for the treatment queue, and saving time for both healthcare professionals and patients [16,23–25]. However, the disadvantages of the telerehabilitation process include the fact that the communication between patients and healthcare professionals may not be well established with telerehabilitation, patients may have difficulties using technological devices for telerehabilitation, technological devices may be broken or internet connection problems may be experienced, patients may have problems understanding the exercises on the screen, and the patients’ activities in treatment sessions or ability to participate in the process are more dependent on their own moods [16,23–25]. First of all, we thought that it would be appropriate to evaluate individuals in order to cognitively manage telerehabilitation. We used the MoCA scale for the evaluation and concluded that all enrolled individuals had scores above 21 points and thus had the cognitive ability to participate in the telerehabilitation program [18]. No participants left the telerehabilitation program over the course of 8 weeks and all participants successfully completed the program. Because we aimed for the telerehabilitation to be successful, we communicated with individual participants one-on-one in each session, repeating and demonstrating the exercises until they understood and performed them correctly. We also sent exercise videos to these patients. With these efforts, we strengthened the communication between the patients and the physiotherapist. However, certain physiotherapy techniques that involve hands-on manipulation or specialized tools are not feasible through telerehabilitation. In our study, the telerehabilitation program primarily comprised exercise regimens with active involvement by both physiotherapists and patients. We believe that exercise constitutes an important treatment approach within physical therapy and rehabilitation that can be consistently maintained for patients. While exercise studies involving telerehabilitation have gained popularity in the literature, previous works such as those of Özlü et al. [26], Shah et al. [16], and Özel and Kaya Ciddi [27] reported only minimal clinically significant improvements in neck pain intensity and disability scores following telerehabilitation exercise programs. In contrast, our findings reveal a noteworthy reduction in neck pain intensity of 4.61 units in the TRG and 4.76 units in the CG following treatment. Additionally, disability scores exhibited a significant median reduction of 7 points in the TRG and CG alike. Our results also align with the minimal clinically significant thresholds for both neck pain intensity (ranging between 1.5 and 2.5 units) and disability scores (between 3.5 and 7.5 points) [28–30].

There was no difference between the groups in terms of neck pain intensity or disability following treatment. This finding confirms that SSE programs administered via telerehabilitation can serve as viable and effective alternatives when in-person exercises are not feasible.

Deep neck muscles provide continuity in the lumbar region, including the M.TrA, the M.LM, and the internal oblique, diaphragm, and pelvic floor muscles. In the literature, it has been reported that changes in motor control activation in the deep trunk muscles are observed in individuals with neck pain [3]. In addition, it has been reported that the activation performance of deep cervical flexors is impaired in patients with low back pain, as in patients with neck pain [31]. Based on this information, the motor relationship between the cervical and lumbar spine regions was investigated to determine whether the muscles in the lumbar region showed similar motor control activation changes in patients with neck pain [3]. Yalcinkaya et al. found that individuals with CNP had less motor control in the M.TrA during abdominal hollowing and rest compared to healthy controls [3]. In another study, Yalcinkaya et al. showed a relationship between right and left upper trapezius muscle pressure pain thresholds and M.TrA thickness during abdominal hollowing in female patients with neck pain [32]. Pinto et al. stated that pain in the neck region may cause a greater response in lower spine regions such as the thoracic and lumbar regions [4]. Yalcinkaya et al. reported that the thoracolumbar fascia and the entire spine are responsible for the stabilization of the M.TrA and the control of rotational movements, and that the average pain intensity of 5.5 according to a VAS in their study may have been effective in this situation [32]. In our study, it was observed that mean neck pain intensity decreased from 7.15 to 2.53 in the TRG and from 6.38 to 1.61 in the CG. Although our results support the results of Yalcinkaya et al. [32], we think that the increase in M.TrA thickness and decrease in neck pain intensity with the SSE program may have increased the stabilization of the lumbar region and helped decrease the large rotational responses. Moseley investigated the relationship between the weakening of trunk muscle functions and increased risk of low back pain in patients with neck pain and followed those patients for 2 years [33]. With the abdominal hollowing maneuver, the probability of developing low back pain was found to be 3–6 times higher both in individuals with neck pain and healthy individuals who showed abnormal responses with a stabilizer biofeedback device compared to those who responded normally. Therefore, abdominal hollowing performance has been reported as a determinant of low back pain development in patients with neck pain [33]. We did not use a stabilizer biofeedback device in our study, but we think that the fact that the lumbar region muscles of patients with neck pain were weakened and the thickness of the muscles increased after the exercise program was objectively demonstrated by ultrasonography, contributing to the literature.

In the literature, it is striking that there are insufficient studies evaluating the effectiveness of lumbar region muscle thicknesses after SSE programs for individuals with neck pain. However, considering the results obtained from individuals with low back pain, Hlaing et al. applied a 4-week SSE program and a strengthening exercise program for individuals with subacute low back pain and found significant changes in M.TrA and M.LM muscle thicknesses in the SSE program compared to the strengthening group [13]. Although Zielinski et al. stated that M.LM thickness was not a predictive factor for individuals to benefit from stabilization exercises [34], M.LM and M.TrA dimensions were reported as predictive factors for the development of low back pain in a systematic review [35]. Although opinions in the literature are thus mixed, it was seen in our results that an 8-week SSE program supported the development of spinal stabilization by increasing the lumbar region muscle thickness in individuals with neck pain. In order to prevent future low back pain in individuals with neck pain, it may be recommended to include individuals in spine-protective stabilization exercise programs and to evaluate the muscle architecture of the lumbar region in order to obtain more clinical findings.

This study has several limitations. Notably, the technological proficiency of patients, a crucial factor for successful telerehabilitation utilization, was not systematically assessed. Given the variability in patients’ technological skills, we experienced initial session delays as participants readied themselves for the exercises. To enhance the participants’ engagement and ease of navigating the process, integrating technology literacy training before the commencement of the exercise program might offer a more productive experience in telerehabilitation programs, especially in addressing practical challenges. Another limitation pertains to the usage of ultrasound equipment, which demands careful skin contact during measurements. This requirement for precision could have introduced minor discrepancies in the test/retest evaluations. Additionally, follow-up of the exercise program’s effects in the subsequent months was lacking, reflecting another limitation of our study.

## 5. Conclusion

In this study, SSEs provided significant and similar improvements in neck pain intensity, disability, and lumbar region muscle architecture when applied remotely with telerehabilitation or face-to-face. Strengthening the muscles of the lumbar region is important to prevent future low back pain in patients with neck pain. In addition, exercises applied with telerehabilitation are an effective alternative that can be used in cases where face-to-face exercises cannot be applied, during pandemics or natural disasters, or for patients who cannot easily access clinics. There is no previous study in the literature that addresses SSEs performed remotely with telerehabilitation in patients with neck pain and effects on the muscle architecture of the lumbar region. Therefore, we anticipate that the results of our study will contribute to the literature.

## Supplementary Information

Figure S1 The 8 weeks spinal stabilization exercise program.

Figure S2 The ultrasonography evaluations of the m. transversus abdominis and m. lumbal multifidus.

## Figures and Tables

**Figure:**
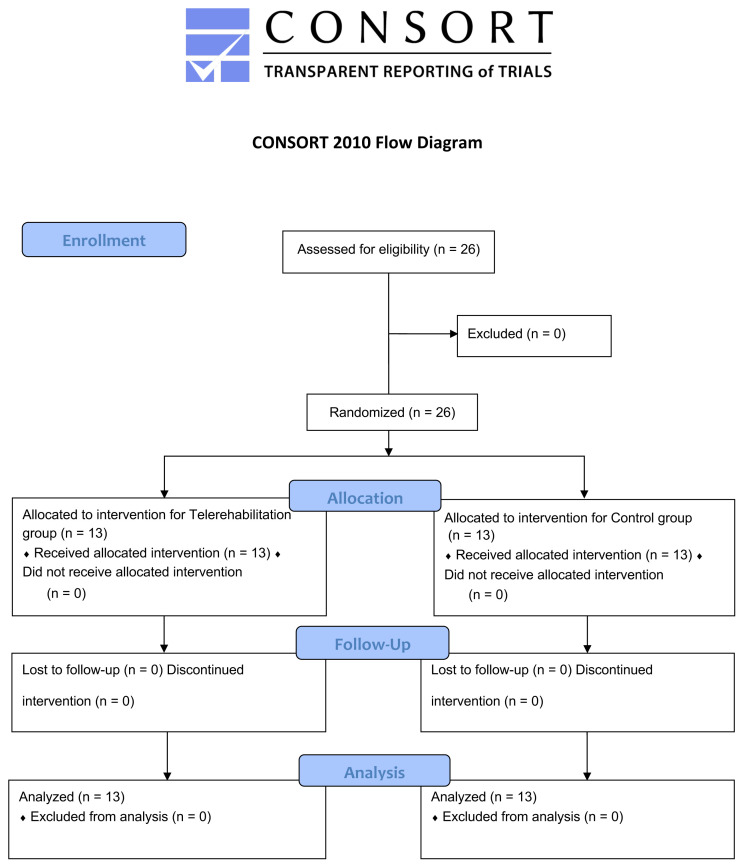
Consolidated Standards of Reporting Trials (CONSORT) flow diagram.

**Table 1:** CONSORT 2010 checklist of information to include when reporting a randomized trial.

Section/topic	Item no.	Checklist item	Reported on page no.
**Title and abstract**			
	1a	Identification as a randomized trial in the title	1
	1b	Structured summary of trial design, methods, results, and conclusions (for specific guidance, see CONSORT for abstracts)	1
**Introduction**			
Background and objectives	2a	Scientific background and explanation of rationale	1, 2
	2b	Specific objectives or hypotheses	2
**Methods**			
Trial design	3a	Description of trial design (such as parallel, factorial) including allocation ratio	2, 5
Trial design	3b	Important changes to methods after trial commencement (such as eligibility criteria), with reasons	2
Participants	4a	Eligibility criteria for participants	2
	4b	Settings and locations where the data were collected	2, 4
Interventions	5	Interventions for each group with sufficient details to allow replication, including how and when they were actually administered	4
Outcomes	6a	Completely defined prespecified primary and secondary outcome measures, including how and when they were assessed	4
	6b	Any changes to trial outcomes after the trial commenced, with reasons	-
Sample size	7a	How sample size was determined	4, 5
	7b	When applicable, explanation of any interim analyses and stopping guidelines	-
Randomization			
Sequence generation	8a	Method used to generate the random allocation sequence	5
	8b	Type of randomization; details of any restriction (such as blocking and block size)	5
Allocation concealment mechanism	9	Mechanism used to implement the random allocation sequence (such as sequentially numbered containers), describing any steps taken to conceal the sequence until interventions were assigned	5
Implementation	10	Who generated the random allocation sequence, who enrolled participants, and who assigned participants to interventions	5
Blinding	11a	If done, who was blinded after assignment to interventions (for example, participants, care providers, those assessing outcomes) and how	5
	11b	If relevant, description of the similarity of interventions	-
Statistical methods	12a	Statistical methods used to compare groups for primary and secondary outcomes	5, 6
	12b	Methods for additional analyses, such as subgroup analyses and adjusted analyses	5, 6
**Results**			
Participant flow (a diagram is strongly recommended)	13a	For each group, numbers of participants who were randomly assigned, received intended treatment, and were analyzed for the primary outcome	5
	13b	For each group, losses and exclusions after randomization, together with reasons	5
Recruitment	14a	Dates defining the periods of recruitment and follow-up	4
	14b	Why the trial ended or was stopped	4, 5
Baseline data	15	A table showing baseline demographic and clinical characteristics for each group	6
Numbers analyzed	16	For each group, number of participants (denominator) included in each analysis and whether the analysis was by original assigned groups	6, 7
Outcomes and estimation	17a	For each primary and secondary outcome, results for each group, and the estimated effect size and its precision (such as 95% confidence interval)	6, 7
	17b	For binary outcomes, presentation of both absolute and relative effect sizes is recommended	6, 7
Ancillary analyses	18	Results of any other analyses performed, including subgroup analyses and adjusted analyses, distinguishing prespecified from exploratory	6, 7
Harms	19	All important harms or unintended effects in each group (for specific guidance, see CONSORT for harms)	-
**Discussion**			
Limitations	20	Trial limitations, addressing sources of potential bias, imprecision, and, if relevant, multiplicity of analyses	9
Generalizability	21	Generalizability (external validity, applicability) of the trial findings	8, 9
Interpretation	22	Interpretation consistent with results, balancing benefits and harms, and considering other relevant evidence	8, 9
Other information			
Registration	23	Registration number and name of trial registry	2
Protocol	24	Where the full trial protocol can be accessed, if available	2
Funding	25	Sources of funding and other support (such as supply of drugs), role of funders	9

**Table 2:** Characteristic of the patients.

Variables	Mean ± SD or median (min–max) / n			p
	Total (n = 26)	TRG (n = 13)	CG (n = 13)	
Age, years	36.19 ± 9.98	35.76 ± 10.36	36.61 ± 9.99	0.83
Sex, female/male	18/8	9/4	9/4	1.0
BMI, kg/m^2^	23.57 ± 2.59	23.6 ± 2.85	23.55 ± 2.43	0.96
Pain duration, months	48 (18–240)	36 (18–240)	60 (18–144)	0.05
MoCA score	30 (25–30)	30 (25–30)	30 (29–30)	0.51

BMI: Body mass index; MoCA: Montreal Cognitive Assessment Scale; TRG: telerehabilitation group; CG: control group; SD: standard deviation; min: minimum; max: maximum.

**Table 3:** Results for thickness of the transversus abdominis and lumbar multifidus muscles.

Variables	Time	Mean ± SD or median (min–max)		p (BT)	ES, Cohen’s d
		TRG (n = 13)	CG (n = 13)		
Right M.TrA, resting (mm)	BT	2.58 ± 0.76	2.64 ± 0.55	0.83	0.02
	AT	3.03 ± 0.80	3.22 ± 0.62	0.51	
	Difference	0.44 ± 0.48	0.58 ± 0.34	0.42	
	p (WG)	0.006^++^	0.000^+++^		
	ES, Cohen’s d	0.91	1.70		
Left M.TrA, resting (mm)	BT	2.57 (1.71–4.77)	2.59 (1.66–3.62)	0.81	0.05
	AT	3.16 (1.88–5.57)	3.08 (2.04–4.45)	0.77	
	Difference	0.27 (−0.19 to 2.11)	0.37 (−0.18 to 1.95)	0.77	
	p (WG)	0.007^++^	0.003^++^		
	ES, Cohen’s d	0.74	0.82		
Right M.TrA, contract. (mm)	BT	4.62 ± 1.02	4.82 ± 1.45	0.69	0.10
	AT	5.88 ± 1.12	5.44 ± 1.45	0.39	
	Difference	1.26 ± 0.92	0.62 ± 0.96	0.09	
	p (WG)	0.000^++^	0.038^+^		
	ES, Cohen’s d	1.09	0.37		

**Table 4:** Results of neck pain intensity and neck disability.

Variables	Time	Mean ± SD or median (min–max)		p (BT)	ES, Cohen’s d
		TRG (n = 13)	CG (n = 13)		
Neck pain intensity (VAS)	BT	7.15 ± 1.90	6.38 ± 2.02	0.328	0.002
	AT	2.53 ± 1.76	1.61 ± 1.44	0.157	
	Difference	4.61 ± 1.38	4.76 ± 1.64	0.798	
	p (WG)	0.000+++	0.000+++		
	ES, Cohen’s d	3.34	2.90		
Neck disability (NDI)	BT	13 (6–9)	12 (5–34)	0.625	0.11
	AT	4 (1–19)	6 (1–10)	0.796	
	Difference	7 (0–18)	7 (1–25)	0.551	
	p (WG)	0.002++	0.001++		
	ES, Cohen’s d	0.85	0.88		
Left M.TrA, contract. (mm)	BT	4.94 ± 1.43	4.84 ± 0.95	0.84	0.12
	AT	6.34 ± 1.39	5.39 ± 1.14	0.06	
	Difference	1.39 ± 1.40	0.54 ± 0.90	0.07	
	p (WG)	0.004++	0.05^+^		
	ES, Cohen’s d	0.87	3.84		
Right M.LM, contract. (mm)	BT	24.35 (19.1–20.9)	26.34 (17.5–32.4)	0.62	0.18
	AT	27.84 (23.7–35.7)	27.78 (22.7–35.9)	0.70	
	Difference	2.87 (1.45–13.21)	1.82 (0.13–9.31)	0.34	
	p (WG)	0.002++	0.002++		
	ES, Cohen’s d	0.84	0.86		
Left M.LM, contract. (mm)	BT	23.46 ± 4.47	25.68 ± 5.04	0.24	0.09
	AT	29.31 ± 3.22	28.52 ± 3.18	0.53	
	Difference	5.85 ± 5.60	2.83 ± 4.10	0.12	
	p (WG)	0.003++	0.028^+^		
	ES, Cohen’s d	0.71	0.79		

BT: Before treatment; AT: after treatment; BG: between groups; WG: within groups; ES: effect size; VAS: visual analog scale; NDI: Neck Disability Index; TRG: telerehabilitation group; CG: control group;

+++ p<0.001;

++ p<0.01;

SD: standard deviation; min: minimum; max: maximum.

## References

[1] Pillen S Boon A Van Alfen N Chapter 42 Muscle ultrasound Masdeu JC González RG Handbook of Clinical Neurology Dordrecht, the Netherlands Elsevier 2016 843 853 10.1016/B978-0-444-53486-6.00042-9 27430445

[2] Mallin G Murphy S The effectiveness of a 6-week Pilates programme on outcome measures in a population of chronic neck pain patients: a pilot study Journal of Bodywork and Movement Therapies 2013 17 3 376 384 10.1016/j.jbmt.2013.03.003 23768285

[3] Yalcinkaya G Ozyurek S Kalemci O Sengul Salik Y Comparison of ultrasonographic characteristics of deep abdominal muscles in women with and without chronic neck pain: a case-control study Journal of Musculoskeletal and Neuronal Interactions 2022 22 1 52 61 35234159 PMC8919665

[4] Pinto BL Beaudette SM Graham RB Brown SHM Experimentally induced neck pain causes a decrease in thoracic but not lumbar spine stability Journal of Biomechanics 2019 90 78 83 10.1016/j.jbiomech.2019.04.031 31040023

[5] Rubí-Carnacea F Masbernat-Almenara M Climent-Sanz C Soler-González J García-Escudero M Effectiveness of an exercise intervention based on preactivation of the abdominal transverse muscle in patients with chronic nonspecific low back pain in primary care: a randomized control trial BMC Primary Care 2023 24 1 180 10.1186/s12875-023-02140-3 37674205 PMC10483714

[6] Izraelski J Assessment and treatment of muscle imbalance: the Janda approach Journal of the Canadian Chiropractic Association 2012 56 2 158

[7] Lee JS Kim TH Kim DY Shim JH Lim JY Effects of selective exercise for the deep abdominal muscles and lumbar stabilization exercise on the thickness of the transversus abdominis and postural maintenance Journal of Physical Therapy Science 2015 27 2 367 370 10.1589/jpts.27.367 25729169 PMC4339139

[8] Kashfi P Karimi N Peolsson A Rahnama L The effects of deep neck muscle-specific training versus general exercises on deep neck muscle thickness, pain and disability in patients with chronic non-specific neck pain: protocol for a randomized clinical trial (RCT) BMC Musculoskeletal Disorders 2019 20 1 540 10.1186/s12891-019-2880-x 31727085 PMC6857347

[9] Javanshir K Mohseni-Bandpei MA Rezasoltani A Amiri M Rahgozar M Ultrasonography of longus colli muscle: a reliability study on healthy subjects and patients with chronic neck pain Journal of Bodywork and Movement Therapies 2011 15 1 50 56 10.1016/j.jbmt.2009.07.005 21147418

[10] Amiri-Arimi S Mohseni Bandpei MA Rezasoltani A Javanshir K Biglarian A Measurement of cervical multifidus and longus colli muscle dimensions in patients with cervical radiculopathy and healthy controls using ultrasonography: a reliability study PM&R 2019 11 3 236 242 10.1016/j.pmrj.2018.07.014 30081216

[11] Noormohammadpour P Dehghani-Firouzabadi A Mansournia MA Mohseni-Bandpei MA Moghaddam N Comparison of the cross-sectional area of longus colli muscle between patients with cervical radicular pain and healthy controls PM&R 2017 9 2 120 126 10.1016/j.pmrj.2016.06.020 27346094

[12] Uluğ N Yılmaz ÖT Kara M Ozcakar L Effects of Pilates and yoga in patients with chronic neck pain: a sonographic study Journal of Rehabilitation Medicine 2018 50 1 80 85 10.2340/16501977-2288 29160551

[13] Hlaing SS Puntumetakul R Khine EE Boucat R Effects of core stabilization exercise and strengthening exercise on proprioception, balance, muscle thickness and pain related outcomes in patients with subacute nonspecific low back pain: a randomized controlled trial BMC Musculoskeletal Disorders 2021 22 1 998 10.1186/s12891-021-04858-6 34847915 PMC8630919

[14] Hosseinifar M Akbari M Behtash H Amiri M Sarrafzadeh J The effects of stabilization and McKenzie exercises on transverse abdominis and multifidus muscle thickness, pain, and disability: a randomized controlled trial in nonspecific chronic low back pain Journal of Physical Therapy Science 2013 25 12 1541 1545 10.1589/jpts.25.1541 24409016 PMC3885835

[15] Beinart NA Goodchild CE Weinman JA Ayis S Godfrey EL Individual and intervention-related factors associated with adherence to home exercise in chronic low back pain: a systematic review Spine Journal 2013 13 12 1940 1950 10.1016/j.spinee.2013.08.027 24169445

[16] Shah N Shetty GM Kanna R Thakur H Efficacy of telerehabilitation for spine pain during the coronavirus pandemic lockdown: a retrospective propensity score-matched analysis Disability and Rehabilitation: Assistive Technology 2024 19 3 558 565 10.1080/17483107.2022.2107718 35930451

[17] Nasreddine ZS Phillips NA Bédirian V Charbonneau S White V The Montreal Cognitive Assessment, MoCA: a brief screening tool for mild cognitive impairment Journal of the American Geriatrics Society 2005 53 4 695 659 10.1111/j.1532-5415.2005.53221.x 15817019

[18] Selekler K Cangöz B Sait U Power of discrimination of Montreal Cognitive Assessment (MOCA) Scale in Turkish patients with mild cognitive impairement and Alzheimer’s disease Turkish Journal of Geriatrics 2010 13 3 166 171

[19] Oz M Ozel Asliyuce Y Demirel A Cetin H Ulger O Determination of cognitive status and influencing variables in patients with chronic neck pain: a cross-sectional study Applied Neuropsychology: Adult 2023 30 6 764 771 10.1080/23279095.2021.1980795 34597197

[20] Price DD McGrath PA Rafii A Buckingham B The validation of visual analogue scales as ratio scale measures for chronic and experimental pain Pain 1983 17 1 45 56 10.1016/0304-3959(83)90126-4 6226917

[21] Telci EA Karaduman A Yakut Y Aras B Simsek IE The cultural adaptation, reliability, and validity of neck disability index in patients with neck pain: a Turkish version study Spine 2009 34 16 1732 1735 10.1097/BRS.0b013e3181ac9055 19770615

[22] Vernon H Mior S The Neck Disability Index: a study of reliability and validity Journal of Manipulative & Physiological Therapeutics 1991 14 7 409 415 1834753

[23] Rabinovitch BS Diaz PL Langleben AC Katz TM Gordon T Wait times and patient throughput after the implementation of a novel model of virtual care in an outpatient neurology clinic: a retrospective analysis Journal of Telemedicine and Telecare (in press) 10.1177/1357633X221139558 36529888

[24] Gali K Joshi S Hueneke S Katzenbach A Radecki L Barriers, access and management of paediatric epilepsy with telehealth Journal of Telemedicine and Telecare 2022 28 3 213 223 10.1177/1357633X20969531 33183129 PMC8980450

[25] Martínez de la Cal J Fernández-Sánchez M Matarán-Peñarrocha GA Hurley DA Castro-Sánchez AM Physical therapists’ opinion of e-health treatment of chronic low back pain International Journal of Environmental Research Public Health 2021 18 4 1889 10.3390/ijerph18041889 33669249 PMC7919815

[26] Özlü A Ünver G Tuna H Erdoğan A Effects of interactive telerehabilitation practices in office workers with chronic nonspecific neck pain: randomized controlled study Telemedicine and e-Health 2023 30 2 438 447 10.1089/tmj.2023.0018 37498517

[27] Özel M Kaya Ciddi P The effectiveness of telerehabilitation-based structured exercise therapy for chronic nonspecific neck pain: a randomized controlled trial Journal of Telemedicine and Telecare (in press) 10.1177/1357633X221095782 35570728

[28] Lauche R Langhorst J Dobos GJ Cramer H Clinically meaningful differences in pain, disability and quality of life for chronic nonspecific neck pain - a reanalysis of 4 randomized controlled trials of cupping therapy Complementary Therapies in Medicine 2013 21 4 342 347 10.1016/j.ctim.2013.04.005 23876565

[29] Kovacs FM Abraira V Royuela A Corcoll J Alegre L Minimum detectable and minimal clinically important changes for pain in patients with nonspecific neck pain BMC Musculoskeletal Disorders 2008 9 43 10.1186/1471-2474-9-43 18402665 PMC2375888

[30] MacDowall A Skeppholm M Robinson Y Olerud C Validation of the visual analog scale in the cervical spine Journal of Neurosurgery: Spine 2018 28 3 227 235 10.3171/2017.5.SPINE1732 29243996

[31] Thongprasert C Kanlayanaphotporn R Abnormal performance of cervical stabilizer muscles in individuals with low back pain Journal of Manual & Manipulative Therapy 2019 27 4 215 221 10.1080/10669817.2018.1560946 30935334 PMC7025690

[32] Yalcinkaya G Sengul YS Ozyurek S Kirmizi M Kalemci O Is the pain pressure threshold linked to the transversus abdominis in women with chronic neck pain?: a preliminary report Somatosensory & Motor Research 2021 38 2 133 139 10.1080/08990220.2021.1879776 33632060

[33] Moseley GL Impaired trunk muscle function in sub-acute neck pain: etiologic in the subsequent development of low back pain? Manual Therapy 2004 9 3 157 163 10.1016/j.math.2004.03.002 15245710

[34] Zielinski KA Henry SM Ouellette-Morton RH DeSarno MJ Lumbar multifidus muscle thickness does not predict patients with low back pain who improve with trunk stabilization exercises Archives of Physical Medicine and Rehabilitation 2013 94 6 1132 1138 10.1016/j.apmr.2012.12.001 23228626 PMC3677852

[35] Wong AYL Parent EC Funabashi Stanton TR Kawchuk GN Do various baseline characteristics of transversus abdominis and lumbar multifidus predict clinical outcomes in nonspecific low back pain? A systematic review Pain 2013 154 12 2589 2602 10.1016/j.pain.2013.07.010 23867731

