# Supplementary figures and images for: Spinal stabilization exercises for transversus abdominis and lumbar multifidus thickness via telerehabilitation and face-to-face approaches in patients with nonspecific chronic neck pain: a randomized controlled trial
Source: Turk J Med Sci. 2024 Jul 12;54(4):811–21. doi: 10.55730/1300-0144.5853 (PMC11407346; doi:10.55730/1300-0144.5853)

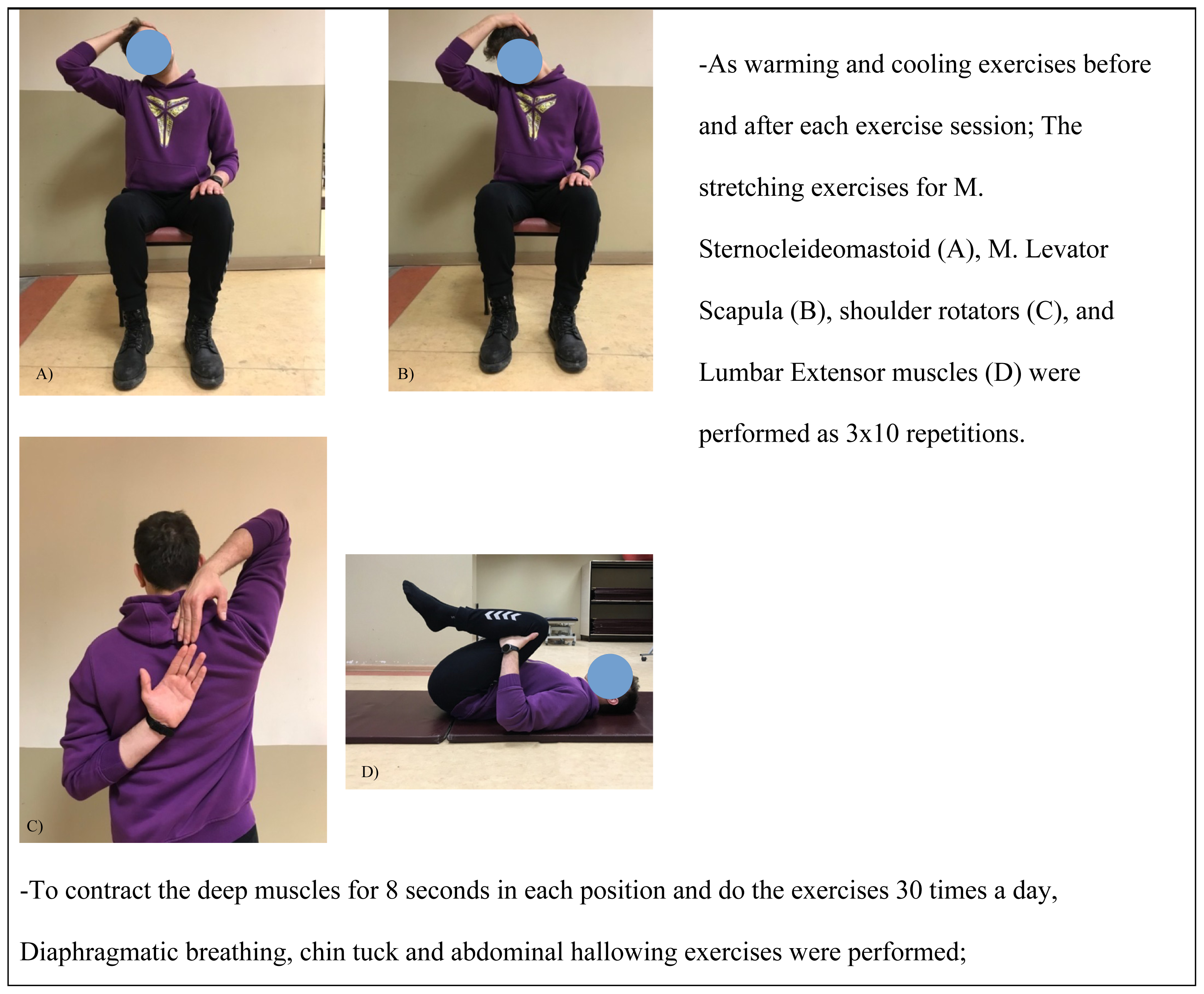

Supplement: Figure S1 — The 8 weeks spinal stabilization exercise program. [file tjmed-54-04-811s1a.tif]

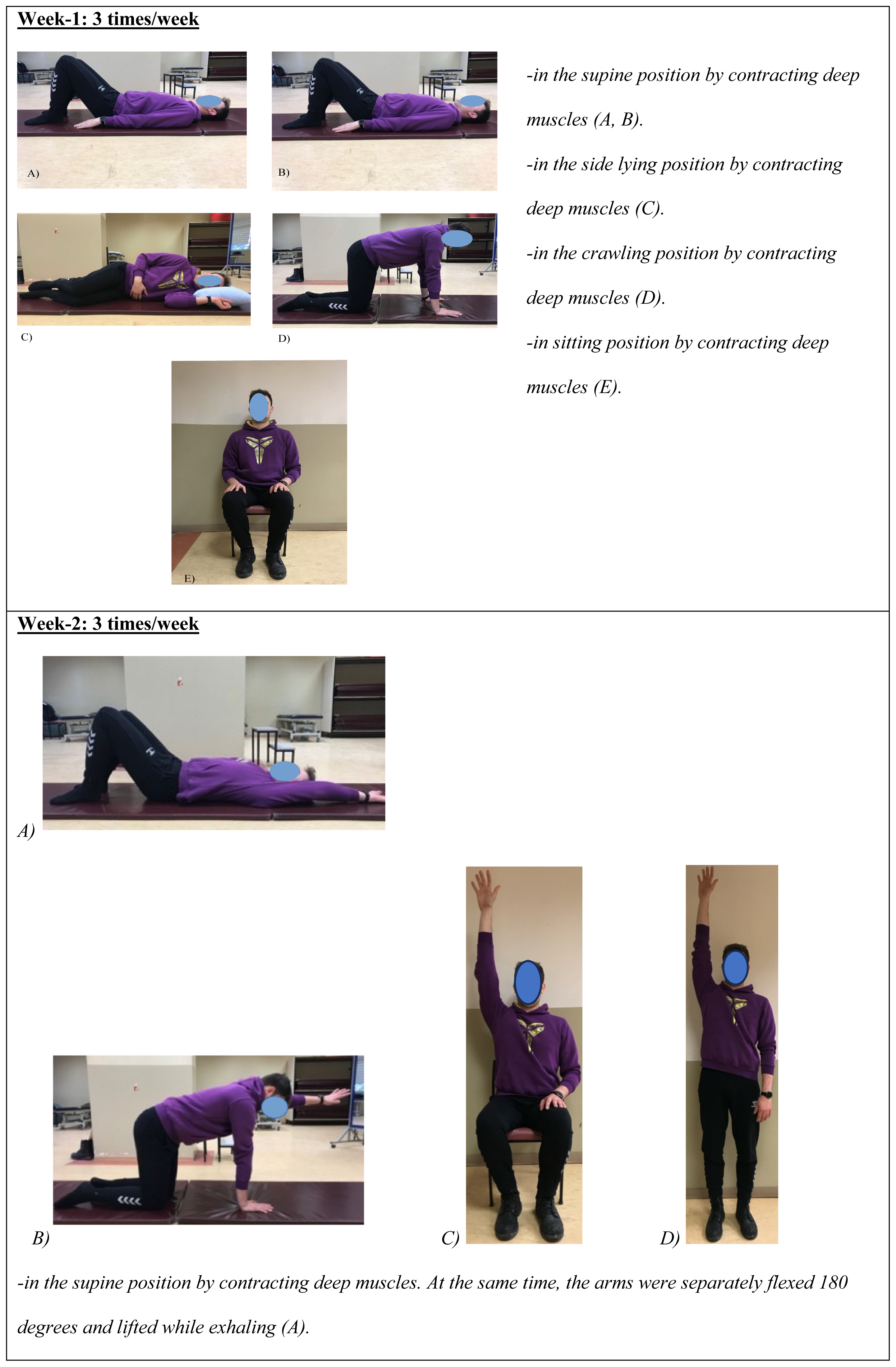

Supplement: Figure S1 — The 8 weeks spinal stabilization exercise program. [file tjmed-54-04-811s1b.tif]

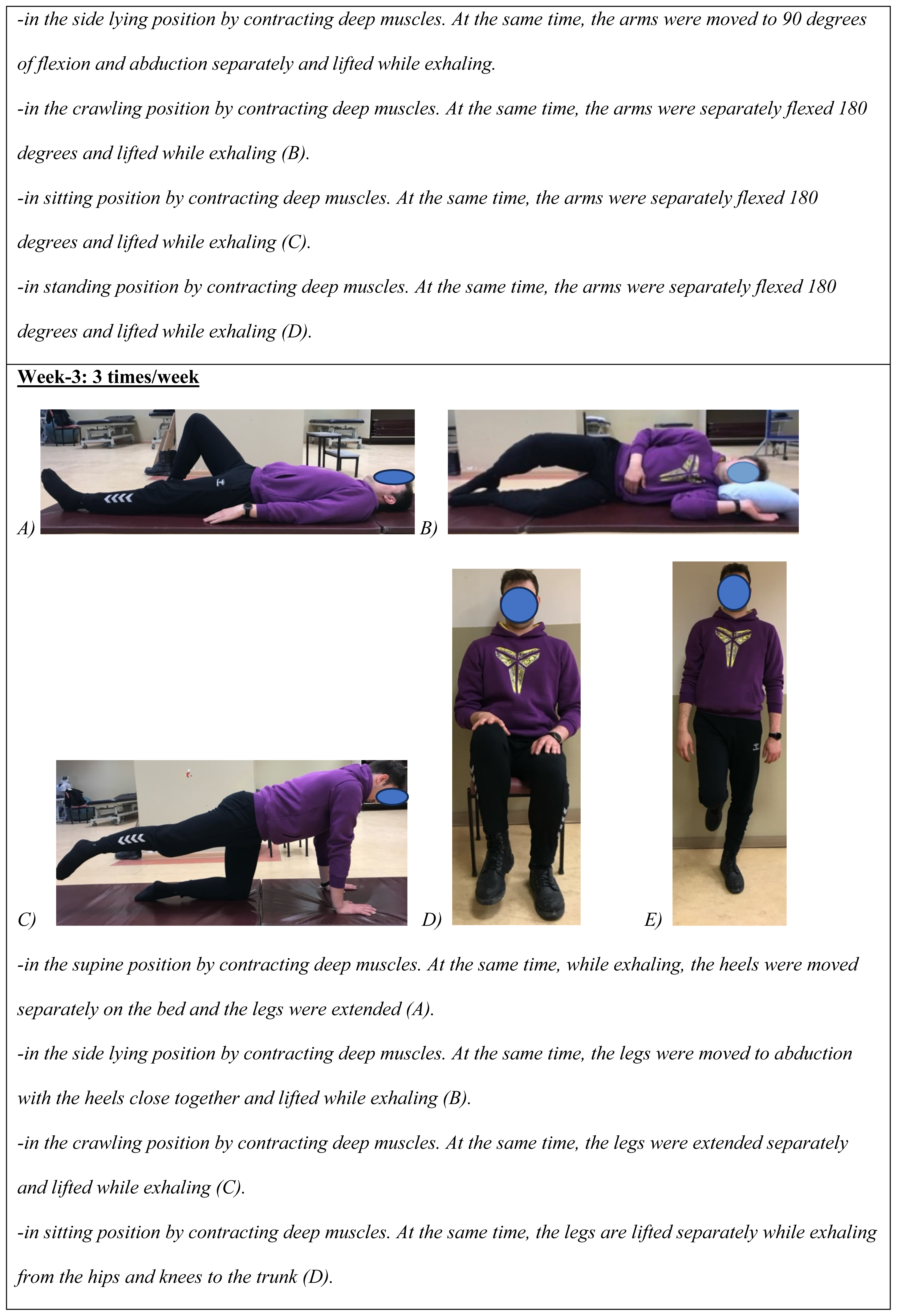

Supplement: Figure S1 — The 8 weeks spinal stabilization exercise program. [file tjmed-54-04-811s1c.tif]

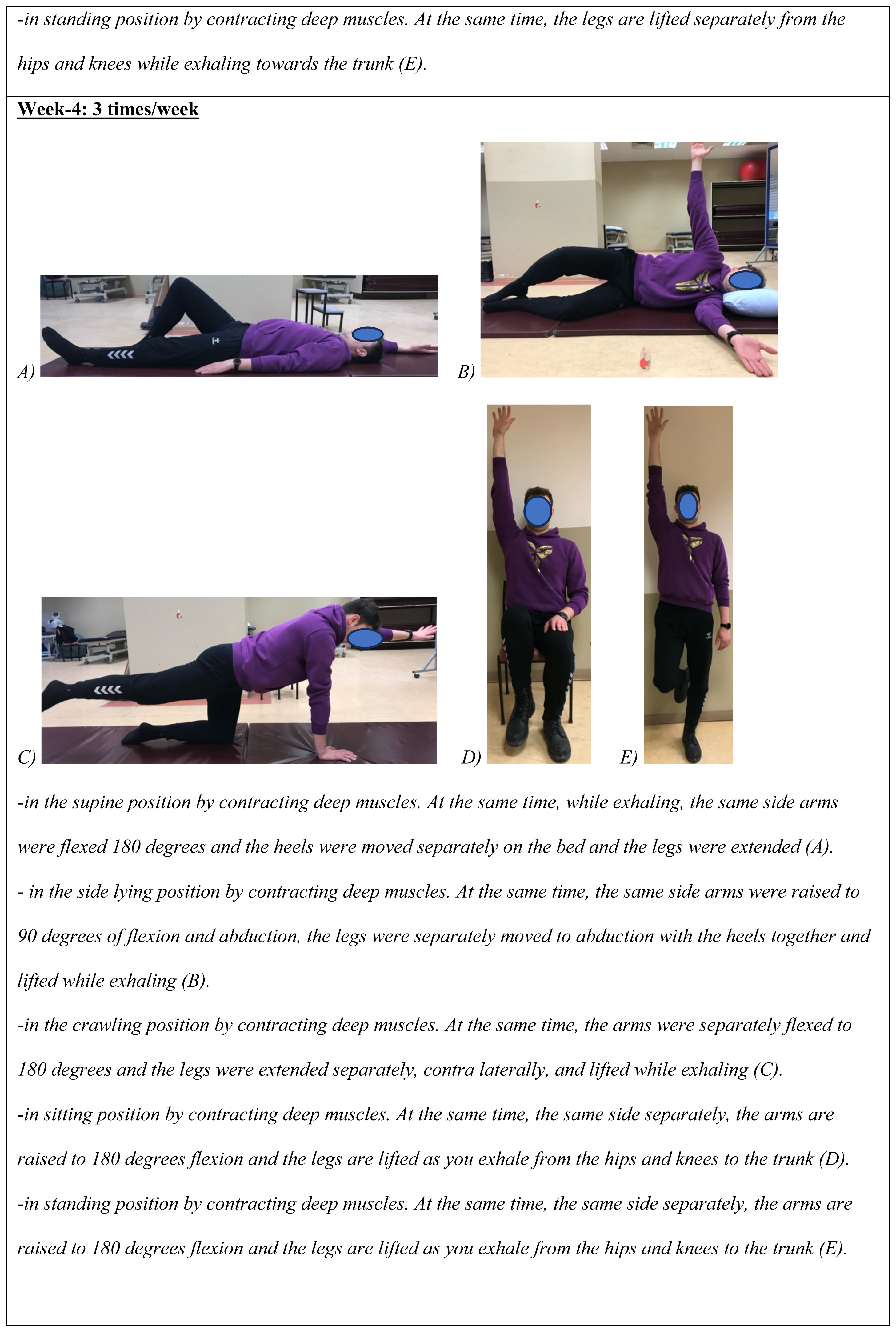

Supplement: Figure S1 — The 8 weeks spinal stabilization exercise program. [file tjmed-54-04-811s1d.tif]

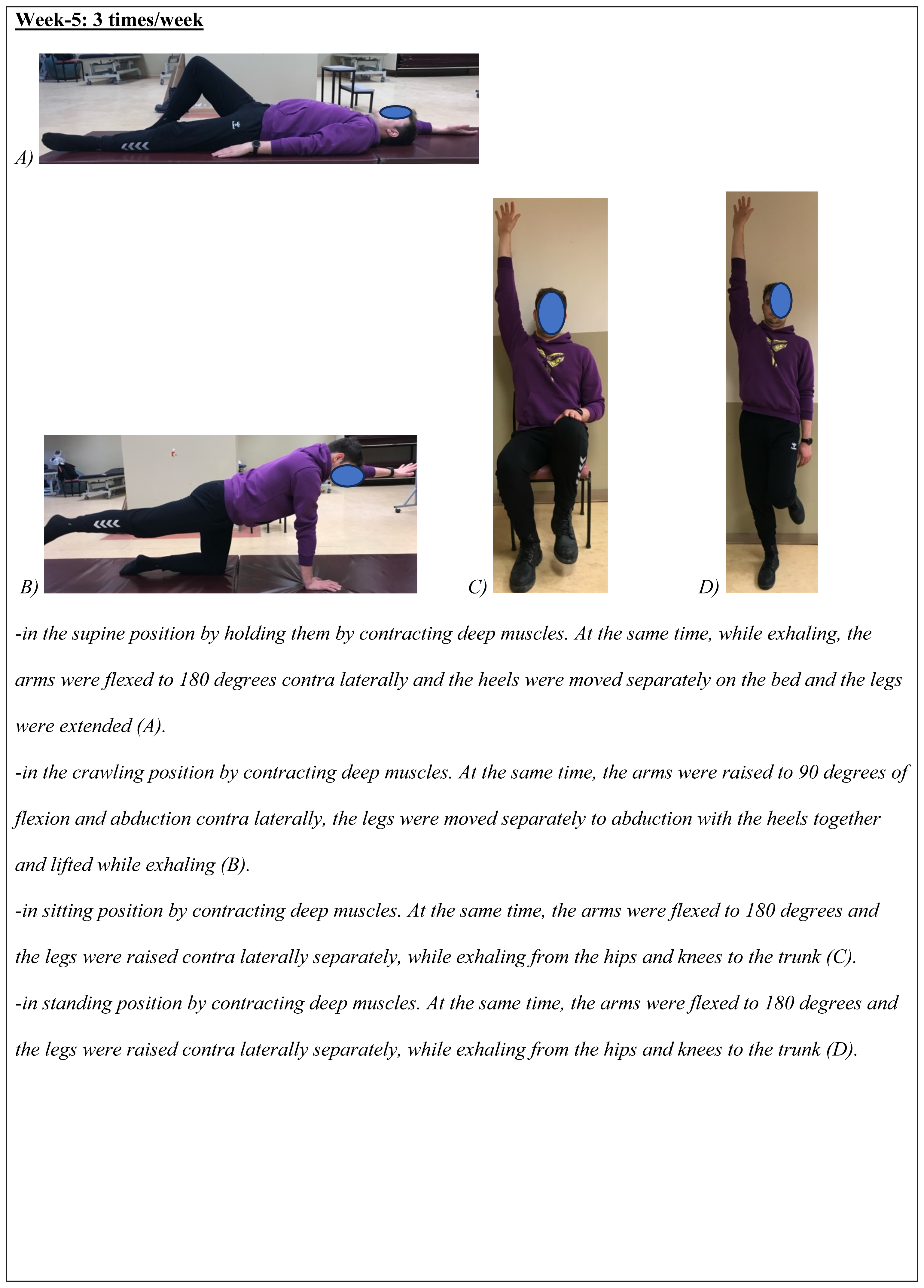

Supplement: Figure S1 — The 8 weeks spinal stabilization exercise program. [file tjmed-54-04-811s1e.tif]

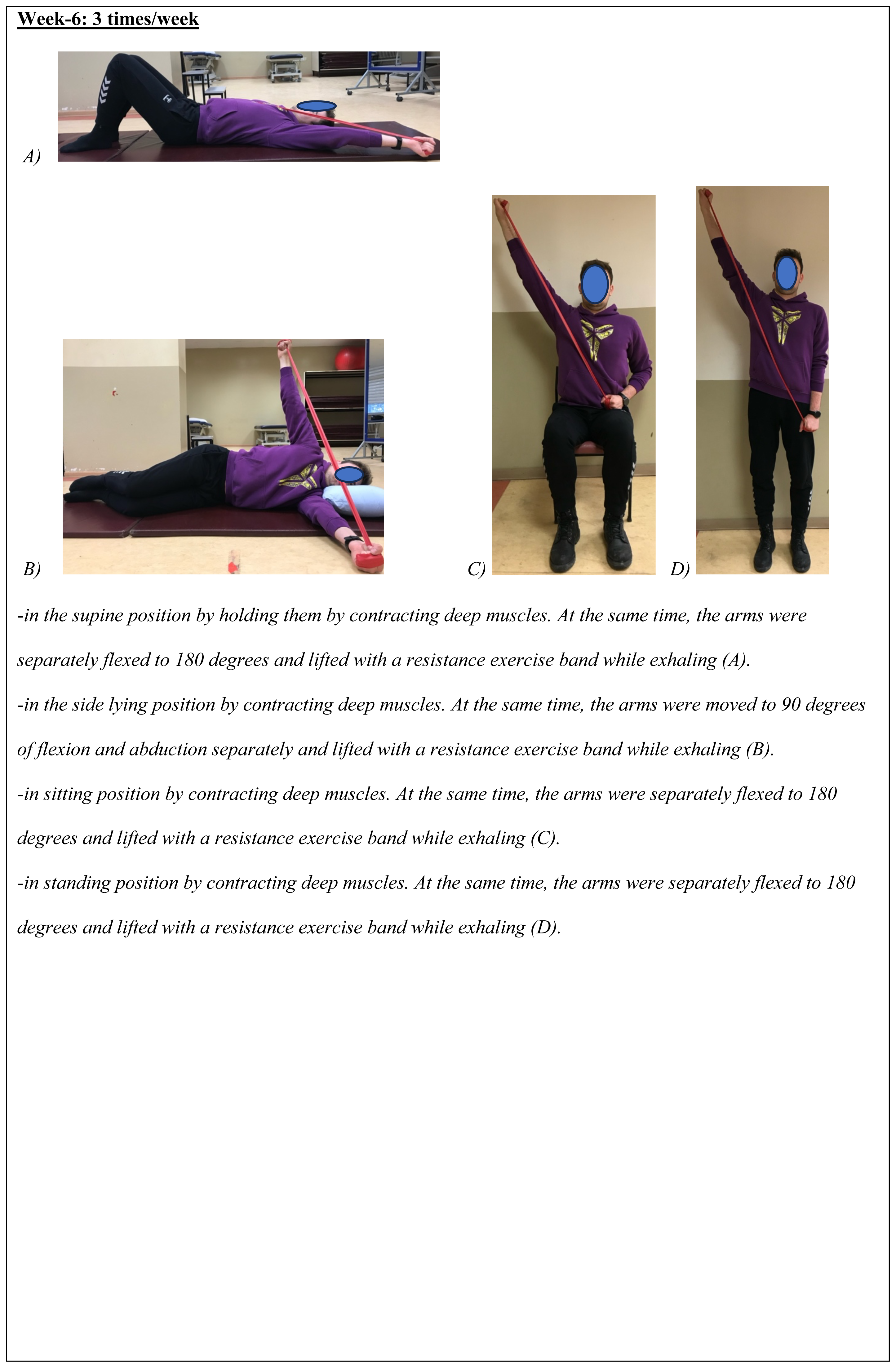

Supplement: Figure S1 — The 8 weeks spinal stabilization exercise program. [file tjmed-54-04-811s1f.tif]

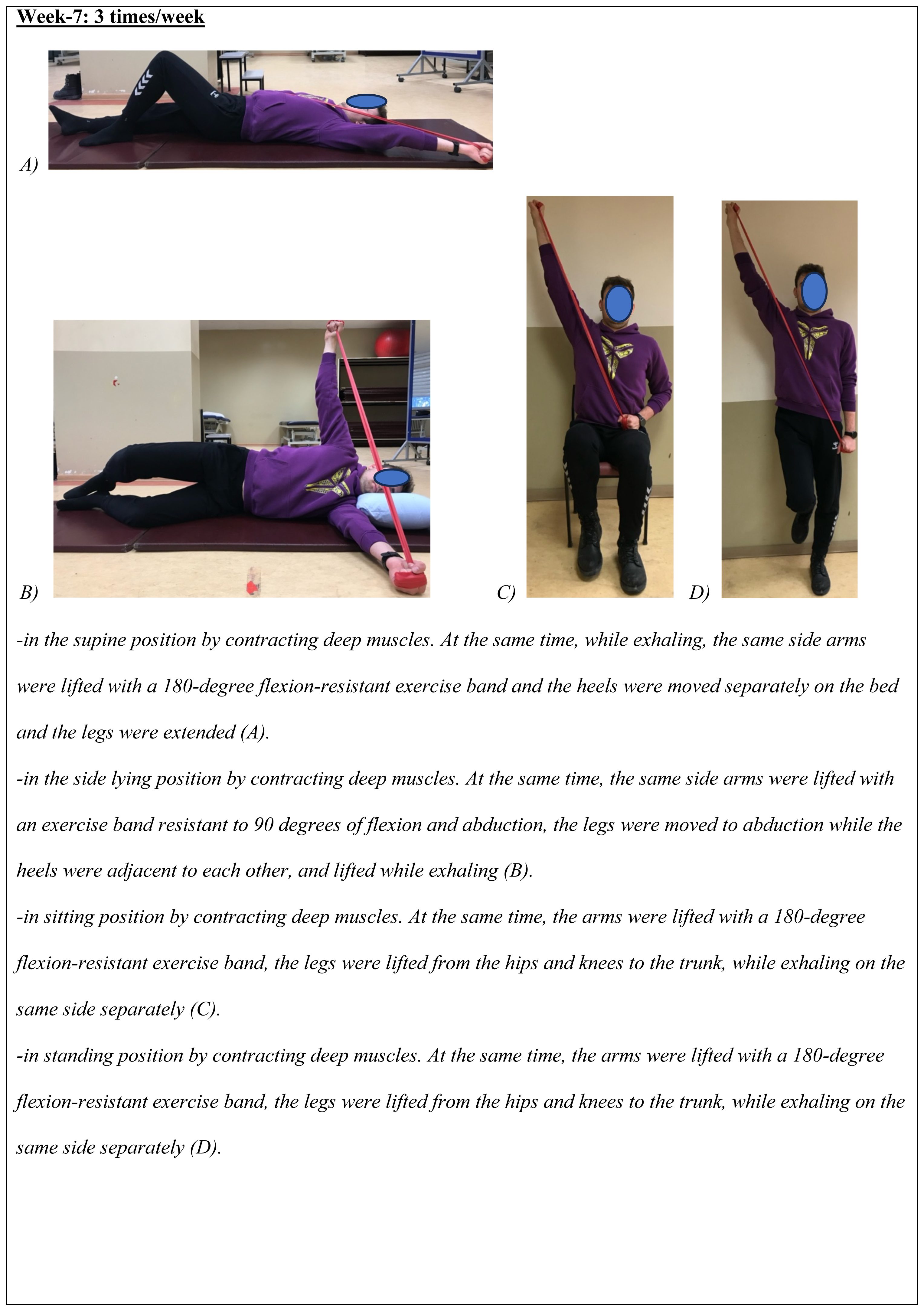

Supplement: Figure S1 — The 8 weeks spinal stabilization exercise program. [file tjmed-54-04-811s1g.tif]

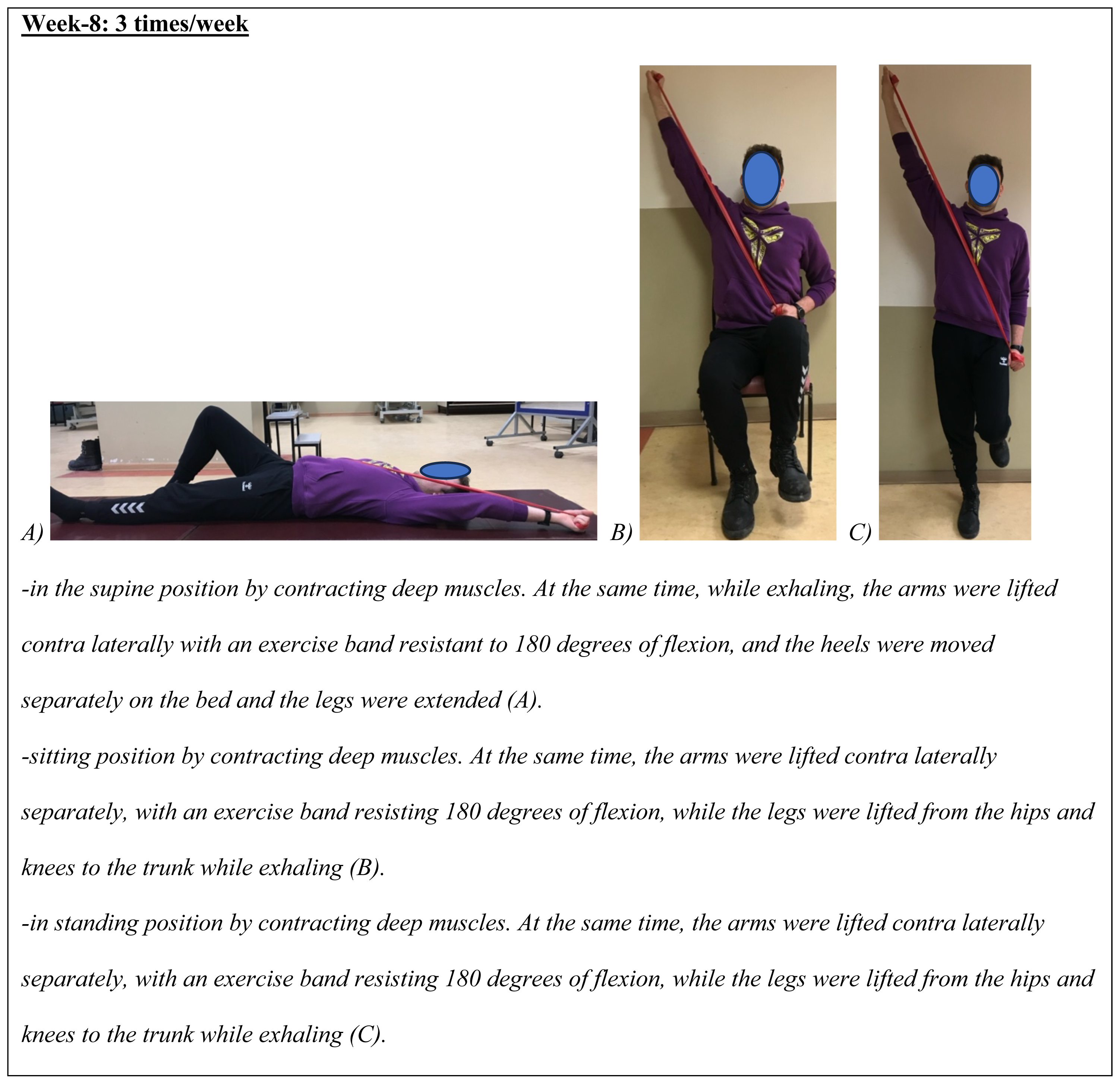

Supplement: Figure S1 — The 8 weeks spinal stabilization exercise program. [file tjmed-54-04-811s1h.tif]

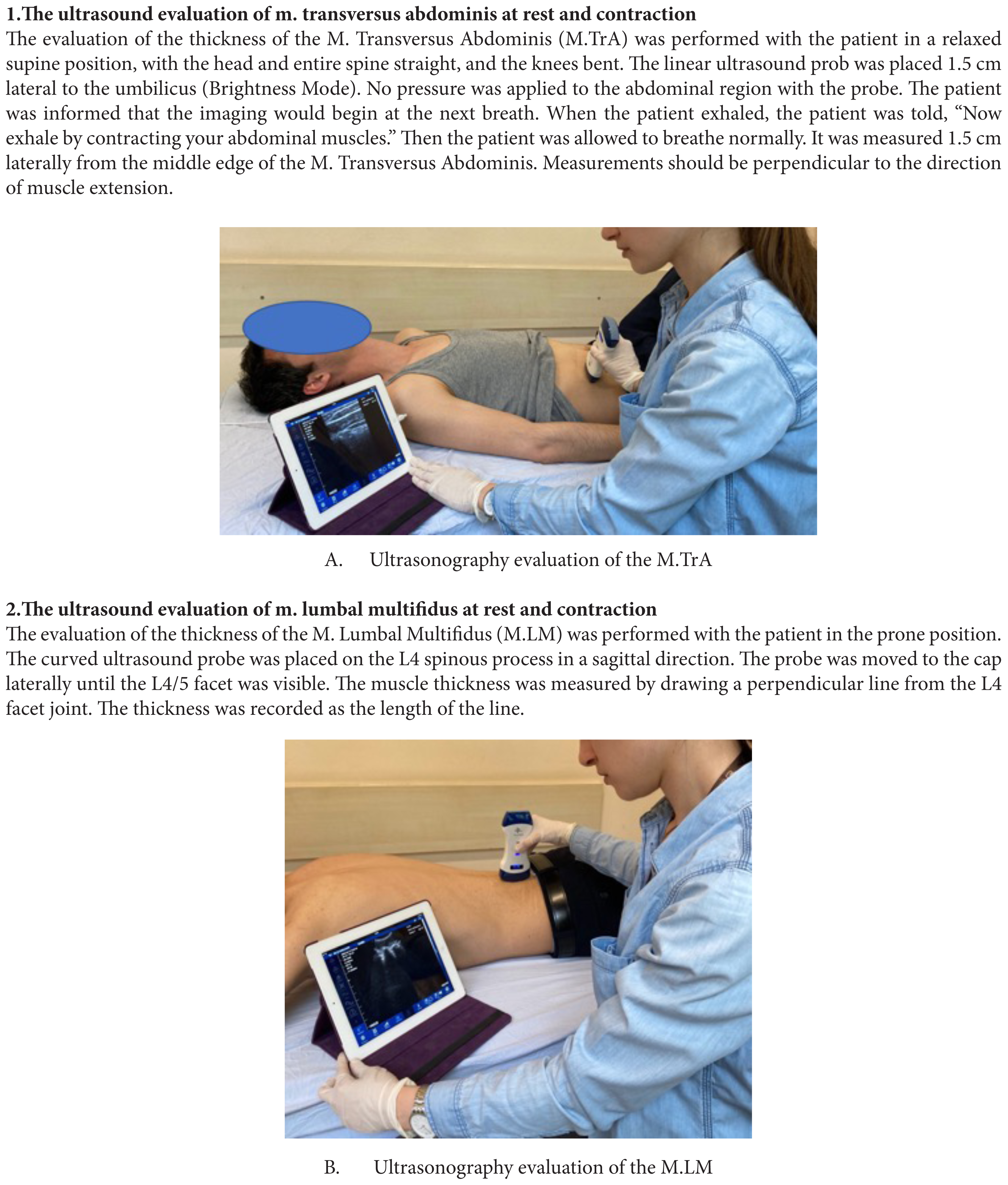

Supplement: Figure S2 — The ultrasonography evaluations of the m. transversus abdominis and m. lumbal multifidus. [file tjmed-54-04-811s2.tif]
